# Effects of adolescent exposure to behaviour change interventions on their HIV risk reduction in Northern Malawi: a situation analysis

**DOI:** 10.1080/17290376.2018.1529612

**Published:** 2018-10-02

**Authors:** M. Mwale, A. S. Muula

**Affiliations:** a Department of Public Health, School of Public Health and Family Medicine, University of Malawi College of Medicine, Blantyre, Malawi; b Department of Education Foundations, Mzuzu University, Mzuzu, Malawi; c Africa Center of Excellence in Public Health and Herbal Medicine, University of Malawi, Zomba, Malawi

**Keywords:** Adolescent, risk reduction, intervention, BCI, HIV and AIDS, Malawi

## Abstract

Understanding adolescents’ translation of HIV and AIDS-related behaviour change interventions (BCI) knowledge and skills into expected behavioural outcomes helps us appreciate behaviour change dynamics among young people and informs evidence-based programming. We explored the effects of adolescents’ exposure to BCI on their HIV risk reduction in selected schools in Nkhatabay and Mzimba districts and Mzuzu city in Northern Malawi. The study used questionnaires as instruments. Data were collected between January and April 2017. Adolescent boys and girls [*n* = 552], ages 11–19 were randomly sampled to participate. Data analysis was through multiple regression and content analysis. Respondents included 324 female [58.7%] and 228 male [41.3%]. Multiple regression analysis indicated that exposure to BCI did not affect risk reduction in the study area. The best stepwise model isolated sexual experience ([Beta = .727, *p* = .0001, *p* < .05]) as having the strongest correlation with the dependent variable – risk reduction. BCI exposure was stepwise excluded ([Beta = −.082, *p* = .053, *p* > .05]). There was therefore no evidence against the null hypothesis of no relationship between adolescent exposure to BCI and their HIV risk reduction. Overall there was limited BCI knowledge and skills translation to behavioural risk reduction. The study points to the need to evaluate and redesign adolescent BCI in line with current behavioural dynamics among young people in Malawi. The findings have been used to inform the design and programming of a model to be tested for feasibility through a quasi-experiment in the second phase of our project.

## Introduction

Malawi, with an estimated HIV prevalence of 10.6% of adults aged 15–49 years is among the 10 countries with the highest HIV prevalence in the world (Malawi Demographic and Health Survey [DHS], 2010; National AIDS Commission [NAC], 2012). Young people especially girls are considered one of the at risk groups mainly due to socio-economic correlates such as poverty and gender disparities (Baxter & Abdool Karim, 2016; Mwale & Muula, 2017; UNGASS, 2010; Underwood, Skinner, Osman, & Schwandt, 2011). According to the Malawi Demographic and Health Survey (DHS) (2010), some young people have had sex by age 10 and about 106,000 girls get pregnant every year. The rate of new HIV infections in the country is high in the 15–24 years age category constituting over half of all new HIV infections with female adolescents being disproportionately affected. Translated to prevalence it is estimated that 60% of those infected with HIV are young women who are also at high risk for early and unwanted pregnancy (NAC, 2012). The most recent DHS (2017) indicates that the percentage of young women and men aged 15–24 years who are HIV infected has risen from 3.6% in 2010 to 5.9% by 2015 and is among the highest prevalence in the Southern African sub-region. UNAIDS (2016) reports that adolescent girls, young women and other key populations, particularly in urban areas, continue to bear the highest burden of the epidemic. In 2016, Malawi had 36,000 (31,000–45,000) new HIV infections and 24,000 (20,000–31,000) AIDS-related deaths (UNAIDS, 2016). Earlier UNAIDS and NAC reports show that only about 42% of young people aged 15–24 years in Malawi have accurate knowledge of HIV and AIDS and in 2012 alone about 6700 new infections occurred among adolescents ages 15–19 years which translated to 18 infections on a daily basis (NAC, 2012; UNAIDS, 2015). Further, poor or low access to contraceptives is according to the DHS (2017) contributing to about 106,000 teenage pregnancies annually with effects on HIV incidence. The same report documents that in 2013, 34,000 new HIV infections were recorded among young people, 7400 of which occurring in children aged less than 14 years.

HIV contraction in Malawi is mainly driven by factors such as; transactional sex, intergenerational sex, unprotected sex, sex under the influence of substances and indulgence in negative cultural practices (Blankenship, Friedman, Dworkin, & Mantell, 2006; Mwale, 2008a; Mwale & Muula, 2017; Rashid & Mwale, 2016; Underwood et al., 2011). Previous studies in sub-Saharan Africa and elsewhere have reported that major drivers of infection among young people in general include; risk-taking being part of identity formation which is often combined with lack of youth friendly health services as well as stigma by service providers (Baird et al., 2012; Barden-O’Fallon et al., 2004; UNAIDS, 2015). Studies have also pointed to onset of sexual activity that is often associated with a lack of knowledge and skills with which to make health choices (Dinkerton, 2001; Doyle, Mavedzenge, Plummer, & Ross, 2012; Mwale, 2008b). Helleringer and Kohler (2007) contend further that adolescence is characterised by patterns of thinking in which immediate gratification tends to take priority over long-term implications. Other studies highlight theoretical factors as contributing to the infection gap among young people. The social comparison bias and optimistic bias that render adolescents to go beyond realistic appraisals and fall prey to positive illusions for instance underscore some theoretical explanations of adolescent vulnerability to HIV infection (Kenyon, Tsoumanis, & Swartz, 2016; Morris & Rushwan, 2015; Mwale, 2008a; Mwale, 2012). Adolescents in essence are adept to employ denial as a copying mechanism relative to chances of contracting HIV (Mkandawire, Tenkorang, & Luginaah, 2013). Other findings have further attributed adolescent susceptibility and sexual risk-taking to poverty and widespread culturally based beliefs and behaviours linking male heterosexual intercourse to overall health with implications on multiple and/or concurrent sexual partnerships (Kenyon et al., 2016; Marklam et al., 2013). Vulnerability has in contemporary times also been ascribed to the fact that young people are growing up at a time when AIDS is a treatable disease (Foundation for AIDS Research, 2010). This has had effects on the sense of urgency hitherto associated with HIV and AIDS issues which has turned into generalised public and political complacency. The major gap arising from a majority of these previous studies with respect not only to Malawi but sub-Saharan Africa in general is that of the high incidence of HIV among young people that does not meaningfully reflect their exposure to diverse BCI programmes targeting HIV and AIDS dynamics. BCI knowledge and skills are not significantly translating into expected HIV risk reduction behavioural outcomes. Several questions arise. First, whether it is a question of intervention relevance or whether the problem can be ascribed to implementation bottlenecks? Second, whether the gap can be attributed to structural or sociocultural pitfalls that imbue or militate against intervention effectiveness and effort? Third, whether the problem can be attributed to the adolescent primary beneficiaries themselves with respect to how they socially construct and psychologically represent BCI messages vis-à-vis their vulnerability to HIV infection? Although several studies have endeavored to explore the BCI exposure vis-à-vis HIV incidence gap among adolescents and young people per se; there still remains dearth with respect to considerations based on socio-culturally or ecologically based public health models in combination with cognitive behavioural approaches. In this paper, the objective is to report a situation analysis that was conducted to appraise whether adolescents’ exposure to HIV and AIDS behaviour change interventions [BCI] has an effect on their HIV risk reduction in the Northern region of Malawi.

## Methods

We applied mixed methods in a sequential correlational and predictive design that combined quantitative and qualitative approaches using questionnaires and focus group discussions [FGDs] as data collection tools. We only present quantitative results in this paper. The study was mainly observational and data was collected between January and April 2017 in Nkhatabay and Mzimba districts and Mzuzu city within the Northern region of Malawi. Administratively Malawi is demarcated into three regions; south, centre and north. Each region is further delineated into districts. The Northern region has five districts but the study only targeted two districts namely Nkhatabay and Mzimba on grounds of high HIV incidence among young people and the high prevalence of risk factors for and drivers of infection. Some schools from Mzuzu city were also involved to capture the urban population. Because only school going adolescents were targeted, schools that are located in sites deemed high prone to risk factors whether in rural or urban sites were purposively sampled to participate. The inclusion of rural samples was based on empirical literature from Malawi documenting low levels of knowledge and self-efficacy in rural Malawian youth in general (DHS, 2010; DHS, 2017; NAC, 2015; UNAIDS, 2015). Further, traditional norms, cultural practices and high levels of poverty in rural populations have also been associated with poor intervention outcomes for transactional sex, teenage pregnancies, school dropouts and early marriages among adolescent females in rural Malawi (Dancy et al., 2014; Weinhardt et al., 2016). The urban samples from Mzuzu city were targeted because urban Malawian adolescents have been demonstrated to be at heightened risk for HIV due to the density of sexual networks as well as vulnerability to transactional and intergenerational sexual relationships (National Plan of Action, 2008).

Altogether, 552 respondents ages 10–19 years were involved in the study and of these, 324 (58.7%) were female and 228 (41.3%) were male. Simple random sampling was utilised in selecting adolescent respondents in each of the twelve schools for purposes of equivalence and bias reduction (Creswell & Clark, 2011; Tashakkori & Teddlie, 1998). The twelve schools from which respondents were randomised were purposively selected from the Northern region registry for schools provided for by the Northern Division schools headquarters. The Cochran formula for populations that are large was used for sample size determination because the variability of the adolescent population for the study site was unknown.

Consented respondents filled a 60-minute self-administered questionnaire at each of the twelve research sites. Questionnaires were self-administered. The questionnaire was self-designed but most items are consistent with standard DHS and NAC items for adolescents and young people in Malawi. Specifically, the questionnaire was prioritised because of its time-saving nature and ability to capture diverse variables in a single, well-structured tool. Reliability analysis was conducted with Cronbach alphas being determined for some of the items targeting risk perception and self-efficacy. The questionnaire was also piloted prior to data collection to ensure that items validly captured what they were intended to measure and to also minimise possible ambiguities or misinterpretation by respondents.

We conducted the study with full respect for current statutes and international conventions guiding research with human participants case in point being the Helsinki Declaration and Charter of Fundamental Rights of the European Union. We took account of stipulated guiding research principles as; anonymity, informed consent, confidentiality and other such data management and protection issues in methods design, data collection, and data analysis as well as interpretation. Specifically, respondents signed consent forms before being involved in the study and were briefed of the study objectives before participation. As most of our respondents were adolescents below the age of 18 and hence considered minors in accordance with statutes and ethics, parental and guardian consent was sought and obtained before data collection. All respondents were assured of the utmost confidentiality of their input. They were told not to include their names on the questionnaires and that codes would instead be used to guarantee confidentiality and anonymity. They were also informed that those who wished to withdraw for whatever reasons even after commencing participation were free to do so as participation was voluntary. Ethics approval for the study was granted by the University of Malawi College of Medicine Research and Ethics Committee.

Data analysis involved first descriptive univariate analysis; that included determining means, standard deviations, medians and interquantile range as well as confidence intervals for the means on continuous data for the main explanatory variables. Frequency distributions as well as crosstabulations on main explanatory variables were also computed. Bivariate analysis through linear regression followed as part of preliminary isolation and identification of variables to be included into our multiple regression model, specifically as part of model building. In the linear regression computations, categorical variables considered to possibly predict or even confound the dependent variable were analysed in their recoded binary formats. Variables that significantly correlated with the dependent variable, both continuous and categorical in the one on one linear regression computations were therefore considered for inclusion in multiple regression analysis through the stepwise selection criterion. The stepwise criterion helped us build our multiple regression model or regression equation. As part of hypothesis testing the model also helped us determine whether our main predictor variable exposure to BCI would accurately predict risk reduction hence establishing a relationship or other variables were rather best predictors. Goodness of fit was determined through the coefficient of determination – *R*^2^.

## Results

The results are presented beginning with respondent background information. We then present respondent sexual behaviour and knowledge findings. Measures of sexual behaviour included; respondent sexual experience (whether someone is sexually active or not), age at debut and condom use at debut. Measures of knowledge included; respondent knowledge on modes of HIV transmission, drivers of infection, VCT knowledge, MMC knowledge and strategies for avoiding and reducing the risk of infection. We go further to present multiple regression analysis results. Multiple regression models were employed in hypothesis testing mainly because the study sought to identify and isolate specific correlates mainly driving the incidence gap in the study area. The same was done for purposes of designing a model for HIV risk reduction.

### Respondent background information

The final sample consisted of 552 adolescent respondents aged 10–19 years. Of these 324 were female constituting 58.7% and 228 were male constituting 41.3%. In the early adolescent age category there were 223 [40.4%] respondents and in the late adolescent category there were 329 [59.6%] respondents. The mean and standard deviation for age were ($\bar{x}$ = 15.11; *s* = 1.175). All the socio-demographic characteristics are displayed in Table 1.

**Table 1.** Respondent background characteristics.

*N *=* *522		
Category	Frequency (N)	Percent
*Age*		
10–14	223	40.4
15–19	329	59.6
*Gender*		
Male	228	41.3
Female	324	58.7
*Religion*		
Christian	548	99.3
Muslim	4	0.7
*School location*		
Rural	377	68.3
Urban	175	31.7
*School type*		* *
Secondary	264	47.8
CDSS (Community	288	52.2

### Sexual behaviour and knowledge

With regards to sexual experience, 262 respondents constituting 47.5%, (CI, 43% ≤ *π* ≥ 49%) were sexually active and 290 respondents constituting 52.5% were sexually inexperienced, (CI, 49% ≤ *π* ≥ 57%). Of the adolescent respondents who were sexually active, 189 [34.2%] compared to 73 [13.2%] acknowledged using condoms at sexual debut. Abstinence knowledge was rated at 96.4%, knowledge of partnerships at 58.2%, knowledge of HIV and AIDS dynamics at 89.7%, knowledge of VCT at 93.3% and knowledge of MMC at 31.2%. The mean knowledge score for respondents and standard deviation were, ($\bar{x}$=75.27; *s* = 16.921). Some of the frequencies are displayed in Table 2.

**Table 2.** Respondent sexual behaviour.

Category	Yes	Frequency %	Distribution No	%	Total
Sexual experience	262	47.5	290	52.5	552
Condom use at debut	189	34.2	73	13.2	262
Abstinence knowledge	532	96.4	20	3.6	552
Abstaining	290	52.5	262	47.5	552
Partner knowledge	321	58.2	231	41.8	552
HIV knowledge	495	89.7	57	10.3	552
VCT knowledge	515	93.3	37	6.7	552
VCT uptake	318	57.6	234	42.4	552
VCT intention [for never tested]	169	30.6	62	11.2	231
MMC knowledge	172	31.2	55	10.0	227
MMC uptake	65	11.8	161	29.2	226
MMC intention [for never circumcised]	81	14.7	85	15.4	166
Ever received money/gifts for sex [trans]	34	6.2	518	93.8	552
Some friends ever received money/gifts for sex	243	44.0	309	56.0	552
Have more than 1 sexual partner	142	25.7	410	74.3	552
Some friends have more than 1 sexual partner	298	54.0	254	46.0	552
Ever had sex under alcohol/drug influence	59	10.7	493	89.3	552
Some friends have sex under alcohol/drug influence	223	40.4	329	59.6	552
Ever been involved in culturally related sex	51	9.2	501	90.8	552
Ever used herbs to increase sexual pleasure	34	6.2	518	93.8	552
Some friends ever use herbs to increase sex pleasure	117	21.2	435	78.8	552

Results also show that 121 adolescents had early sexual debut representing 46%, (CI, 41% ≤ *π* ≥ 51%) of those who were sexually active while 141 representing 54%, (CI, 48% ≤ *π* ≥ 59%) had late sexual debut. Of those who had early sexual debut, a majority were from rural settings compared to their urban counterparts. This is displayed in Table 3.

**Table 3.** Respondent cognitions and age at debut by location.

Category	*N*	%
*Risk of HIV in 5 years [self]*		
No risk	242	43.8
Low risk	110	19.9
Moderate risk	32	5.8
High risk	168	30.5
Total	552	100
*Risk of HIV in 5 years [other adolescents]*		
No risk	152	27.5
Low risk	100	18.1
Moderate risk	92	16.7
High risk	208	37.7
Total	552	100
*Number of sexual partners if more than 1*		
2	93	16.8
3	11	2.0
More than 3	39	7.1
Total	143	25.9
*Sexual experience vs location*		
Rural	224	85.4
Urban	38	14.5
*Age at debut*		
10–14 [early adolescence]	121	46.0
15–19 [late adolescence]	141	54.0
Male [earl y debut]	65	54.0
Female [early debut]	56	46.0
*Age at debut vs location*		
Rural early [10–14]	100	82.6
Urban early [10–14]	21	17.3
Rural late [>14]	124	87.9
Urban late [>14]	17	12.0

### Multiple regression analysis and hypothesis testing

Hypothesis testing was through multiple regression analysis. We aimed to test whether adolescent exposure to BCI does affect or predict their HIV risk reduction. Our null hypothesis was that, ‘there is no relationship between adolescent exposure to BCI and HIV risk reduction’. Exposure to BCI being a categorical variable was recoded to binary format (0,1 on Yes or No) to suit our analysis procedure. Risk reduction was measured as a composite index ranging, 0–100, and the mean respondent score on risk reduction and the standard deviation were, ($\bar{x}$ = 58.18, *s* = 24.189 – normally distributed). Our multiple regression procedure included preliminary linear regression on pairs of independent variables. Categorical variables, whether natural confounders like age and gender or other covariates included in one on one linear regression were like the main predictor variable also recoded to binary format. The regression pairing also factored in the dependent variable [risk reduction] and the main predictor independent variable [exposure to BCI]. We then isolated those variables that correlated significantly with the dependent variable which was a step towards model building as displayed in Table 4.

**Table 4.** Pair-wise linear regressed independent variables correlating significantly with the dependent variable.

Independent variable		Dependent variable	Correlation [*r*]	*p*-value [*p *<* *.001]
Sexual experience	vs	Risk reduction	.727	.000
Number of sexual partners	vs	Risk reduction	.643	.000
Culturally related sex	vs	Risk reduction	.366	.000
Knowledge on dynamics	vs	Risk reduction	.357	.000
Transactional sex	vs	Risk reduction	.327	.000
Age at debut	vs	Risk reduction	.195	.000
Involvement in BCI design	vs	Risk reduction	−.123	.000
Abstinence knowledge	vs	Risk reduction	−.190	.000
Exposure to BCI^a^	vs	Risk reduction	−.082	.053

Note: Linear regression analysis was utilised to identify covariates to fit into the multiple regression model together with BCI exposure as part of predictor determination.

^a^Null hypothesis: There is no relationship between adolescent exposure to BCI and HIV risk reduction.

The main predictor variable, exposure to BCI; was noted as not significantly correlating with the dependent variable – risk reduction [*R* = .082, *p* = .053, *p* > .05]. The variables that significantly correlated with the dependent variable in pair-wise linear regression were considered for inclusion in multiple regression model building. The rational was to determine whether they could in aggregate predict or explain risk reduction but also to determine the covariate that best predicted risk reduction. Through the stepwise inclusion and exclusion criteria we built our regression model or equation and the best fitting model isolated sexual experience as having the strongest predictive or explanatory power on risk reduction – the dependent variable, (*X*_1_ [Beta = .727, *p* < .001]). The overall model explained 53% of the variance in risk reduction which was significant (*F*[1,529] = 617.138, *p* < .001]). *R*^2^, the coefficient of determination was utilised to establish goodness of fit. Other variables that formed part of the stepwise inclusion and exclusion criteria at diverse levels included; not having multiple sexual partners [X2], not indulging in sex under alcohol or drug influence [X3], knowledge on HIV and AIDS dynamics [X4], VCT uptake [X5], not indulging in culturally related sex [X6], involvement in BCI programming [X7] and knowledge on abstinence [X8] in the variate:

$$y=b_{0}+b_{1}x_{1}+b_{2}x_{2}+b_{3}x_{3}+b_{4}x_{4}+b_{5}x_{5}+b_{6}x_{6}+b_{7}x_{7}+b_{8}x_{8}$$

where: *b*_0_ is the ‘*y* intercept’, *b*_1_, … *b*_8_ represent the slope of the regression line, *x*_1_, … *x*_8_ represent the independent variables.

One on one correlation between respondents’ knowledge on HIV dynamics and risk reduction indicated a weak positive correlation, [*R* = .357, *p* = .0001, *p* < .05]. Respondent knowledge scores were significantly associated with risk reduction scores but the association was weak. This result points to a weak link between knowledge on HIV dynamics and subsequent HIV risk reduction outcomes and seems to substantiate other findings which indicate contrary to the knowledge base that a significant proportion of respondents in the research site indulge in risky sexual behaviours likely to drive infection. Exposure to BCI was stepwise excluded in our multiple regression analysis as not predicting or explaining adolescent HIV risk reduction. There was therefore no evidence against the null hypothesis of no relationship between BCI exposure and HIV risk reduction. In essence, BCI exposure does not explain or affect HIV risk reduction among adolescents in the research site.

## Discussion

Our study goal was to assess the effects of adolescent exposure to BCI on their HIV risk reduction. HIV risk reduction behaviours include; limiting the number of sexual partners; delaying sexual initiation, practicing abstinence and consistently using condoms (Agha and van Rossem, 2004; Coates, Richter, & Caceres, 2008; Dancy et al, 2014) ). Our main study assumption was that such risk reduction behaviours are a product of BCI exposure and are mainly instilled through knowledge and HIV risk reduction skills building (UNAIDS, 2016). Knowledge and HIV risk reduction skills building have previously been linked to behavioural change (Barden-O’Fallon et al., 2004; Marklam et al., 2013; Mkandawire et al., 2013; Mwale, 2008a; Mwale & Muula, 2017). The desired effect of improving the level of knowledge about AIDS and its prevention according to much of this previous literature is that individuals will become motivated to alter the behaviours that put them at risk for contracting the HIV virus (Underwood et al., 2011).

Our results indicated that a majority of respondents had relatively good knowledge of HIV dynamics [mode of transmission, prevention and care], good knowledge of abstinence and biomedical prevention protocol like VCT. That knowledge, however, was for most adolescents especially in rural settings not consistent with their expected risk reduction outcomes. On aggregate for instance, a relatively high proportion of respondents were sexually active and had initiated sex at an early age. This trend was more pronounced in rural respondents as compared to their urban counterparts. Rural adolescents were also high on the index of those prone to unprotected sex at debut, early sexual debut, multiple partnerships and low-risk perception. This result was striking as we also noted that rural adolescents are mostly neglected when it comes to extra-curricular BCI programmes promoted by private organisations.

Previous studies point to lower skills and efficacy development as well as poverty as being some of the correlates contributing to the HIV incidence gap in rural Malawi (Dancy et al., 2014; UNAIDS, 2015; Underwood et al., 2011; Weinhardt et al., 2016). The findings thus underscore the observation of Merson, O'Malley, and Serwadda (2008) that despite mounting evidence that available behavioural strategies are effective; global prevention efforts remain insufficient, as reflected by key prevention services currently reaching less than 10% of individuals at risk worldwide. Currently there are proposals to emphasise more on structural interventions that target long-lasting changes and policies such as girl empowerment through educational scholarships, free education and social cash transfers (Blankenship et al., 2006; Cluver et al., 2013; Luiz, 2012; Mwale & Muula, 2017; Reed & Miller, 2014). It is hoped through such initiative, interventions may tap at the root distal causes of risky sexual behaviours mainly socio-economic inequalities like poverty and gender disparities with long-term effects especially for rural adolescents relative to HIV risk reduction. In the Malawian scenario one such initiative was the phased out USAID funded GABLE programme that gave scholarships to underprivileged girls especially from rural settings to pursue their secondary education. Another initiative is the CAMFED programme that is being implemented through the Livingstonia Synod AIDS Programme [LISAP] in the Northern region of Malawi, which also provides bursaries for vulnerable and underprivileged girls to pursue their secondary education (CAMFED, 2016; UNICEF, 2015).

The argument for stressing such structural interventions especially for adolescent girls is that social support for schooling is more likely to reduce tendencies for prostitution and sexual work and hence reduce their vulnerability to contracting HIV (Blankenship et al., 2006; Hallet, Gregson, Lewis, Lopman, & Garnett, 2007; Luiz, 2012; Rashid & Mwale, 2016; Underwood et al, 2011). Interventions also need to be tailored in line with age categories. Interventions targeting early adolescents and the very young could focus on abstinence and delayed debut. On the other hand with the observation that late adolescents are more likely to be sexually active especially in rural settings, interventions targeting them may focus more on faithfulness and condom use.

Another striking finding was the role being played by the conformity bias commonly referred to as peer pressure (Kenyon et al., 2016; Mwale, 2012) in motivating risk-taking behaviours that are more likely to result in HIV infection in the study area. Such behaviours as engagement in multiple partnerships, unprotected sex, early initiation of sexual activities, sex under drug and alcohol influence were noted to be mainly determined by peer pressure or the wish to conform to peer norms and values. Therefore, by capitalising on the same peer social networks that act as key pathways through which high-risk sexual behaviours emanate and are perpetrated (Kenyon et al., 2016), we in line with this premise propose more implementation of peer education interventions in schools. This strategy could work best through group dynamics to instil and build HIV risk reduction skills as well as comprehensive knowledge.

There was also a striking pointer to adolescent respondents being subjected to contradictory messages for instance on abstinence, condom use, multiple partnerships and early sexual initiation from diverse sources. Sources of contradictory messages as pointed out include; culture, religion, peers and sometimes interventions. With respect to culture; diverse traditional norms and values were noted to be in conflict with what interventions aim for in HIV risk reduction. There was for instance a pointer towards cultural tolerance of early sexual initiation, low condom use, as well as multiple partnerships in the study area. Diverse previous studies have documented a similar trend across sub-Saharan African countries as one major barrier to intervention efforts and success (Dancy et al., 2014; Kenyon et al., 2016; Mwale, 2008b; Peltzer, 2010; Rashid & Mwale, 2016; Stokl, Karlra, Jacobi, & Watts, 2013; Underwood et al., 2011).

Interventions were also highlighted as another potential source of contradictory messages in the sense that some programmes focused more on condom promotion while others emphasised only abstinence. It was therefore apparent that the abstinence vis-a-vis comprehensive sexuality debate in BCI programming still rages on (De Irala & Alonso, 2006) and is trickling down to primary beneficiaries. Advocates of the abstinence only approach argue that those who emphasise comprehensive sexuality education do not consider seriously that the implementation of ‘A’ – abstinence or ‘B’ – being faithful is possible, incidentally portraying them as unrealistic yet disproportionately focusing on the ‘C’ – condom function. Further it is argued that condom promotion using the ‘safe sex message’ typical of comprehensive sexuality education may actually foster a false sense of security in youth and lead, paradoxically, to increased risk-taking behaviours and vulnerability such as beginning sex at earlier ages and having more sexual partners (Luiz, 2012; Lam, Marteleto, & Rachhod, 2013). This false sense of security is labelled risk compensation by Dinkerton (2001).

While abstinence only approaches have previously been proven to be difficult to implement due to poor outcomes (Morris & Rushwan, 2015; Reed & Miller, 2014; Vermund, Allen, & Abdool Karim, 2009), focusing more on comprehensive sexuality approaches that also factor in faithfulness to one partner and condom use may prove realistic (Vermund et al., 2009). This, however, needs to take cognisance of the mosaic of contextual, sociocultural, socioeconomic and environmental correlates that often militate against intervention efforts and effectiveness. Similar studies in future could therefore consider applying broader and multilevel frameworks such as the ecological, social cognitive and socio-cultural theories (Bandura, 1977; Bronfenbrenner, 1979; Vygotsky, 1978) that factor in environmental, ecological, cultural and contextual determinants of behaviour. An integration of cognitive theory (Fishbein & Ajzen, 1975; Strecher & Rosenstock, 1997) into such ecological and sociological models would also help tap on both contextual and individual determinants of behaviour. The rationale being that, apart from individual factors, sexual behaviour within sub-Saharan African societies is also mainly determined by sociological, cultural and environmental factors that go beyond individual dynamics (Michielsen, Chesich, Temmerman, Dooms, & van Rossem, 2012). We further recommend tailoring interventions in line with location as well as age categories of adolescents. Rural adolescents need not be neglected but considered a special risk group. There is also more that needs to be done to address socioeconomic inequalities and other structural disparities in rural communities if interventions are significantly going to impact significant behavioural change.

## Limitations

The study was mainly conducted as situation analysis to delineate correlates that could be specific to the study area but not general to Malawi. Overall the study served to inform the design and modelling of a quasi-experiment to be rolled out in the same research site. Our study, however, had limitations mainly due to the fact that we used self-reports as our main source of sexual behavioural data. Sexual behaviour, and especially that collected through surveys or questionnaires is prone to respondent bias. Further, the taboo and sensitivity associated with sexuality issues in sub-Saharan African societies subjects sexual behaviour data collection to possible social desirability bias. Respondents in our study might therefore have given responses likely to be deemed socially desirable but not objectively so, just to suit research purposes. Respondents might also have been tempted to underreport or over report their experiences for example male respondents exaggerating their number of sexual partners and female respondents underreporting such. We controlled for this limitation in the framing of our items for the questionnaire to improve on the reliability of the data collection instruments. Instruments were also piloted prior to data collection to ensure they captured what they were meant to measure and to reduce ambiguities and misrepresentations by respondents.

## Conclusion

Overall the study findings contribute to an understanding of how adolescents translate BCI knowledge, skills and messages into subsequent risk reduction behavioural outcomes. The study also helps us predict some proximate and distal barriers to HIV behaviour change. Such understanding can also help inform evidence-based programming and design of sustainable prevention models. The study therefore has implications for adolescent health and HIV risk reduction not only in Malawi but other sub-Saharan African countries with similar generalised epidemics. The main lessons drawn from the study include the need to involve young people in designing interventions that target them. Involving primary adolescent beneficiaries in research programme design can help us to directly tap what determines their vulnerability as well as best strategies to mitigate such. Expert top-down approaches have long been associated with intervention failure and lack of effectiveness in sub-Saharan Africa and elsewhere and our approach serves as a benchmark for beneficiary involvement. Behavioural change is, however, long-term and incremental in nature (Laga, Rugg, Peersman, & Ainsworth, 2012; Maticka-Tyndale, 2012; Michielsen et al., 2012; Ross, 2010; Swartz et al., 2012), therefore while some interventions might not have an immediate impact, positive outcomes might be due but after long periods of time. In Malawi and elsewhere, tailored prevention efforts involving primary beneficiaries themselves need to be promoted. Apart from that there should also be a shift towards structural interventions that aim not only to address proximate determinants of behaviour but rather underlying distal correlates such as poverty and socioeconomic and gender disparities through policy change. It is also pertinent to underscore that involvement of young people in designing and formulating programmes that target them such as those that tap at key social network pathways like peer education may help slow the spread of HIV in the at risk group and further enhance sustainability of interventions.
